# Economic evaluation of dapagliflozin versus metformin in Chinese patients whose diabetes is inadequately controlled with diet and exercise

**DOI:** 10.1186/s12962-020-00208-w

**Published:** 2020-02-28

**Authors:** Hua Nian, Xu Wan, Jing Ma, Fu Jie, Bin Wu

**Affiliations:** 1 Department of Pharmacy, Yueyang Hospital of Integrated Traditional Chinese and Western Medicine, Shanghai University of Traditional Chinese Medicine, Shanghai, China; 2 Medical Decision and Economic Group, Department of Pharmacy, Ren Ji Hospital, South Campus, School of Medicine, Shanghai Jiaotong University, Jiangyue Road 2000, Shanghai, China; 3 Department of Endocrinology, Ren Ji Hospital, South Campus, School of Medicine, Shanghai Jiaotong University, Shanghai, China

**Keywords:** Cost-effectiveness, Dapagliflozin, Metformin, Type 2 diabetes mellitus, Chinese setting

## Abstract

**Background:**

To investigate the long-term economic outcome of dapagliflozin versus metformin in Chinese patients with type 2 diabetes mellitus (T2DM) whose diet and exercise have not provided sufficient glycemic control.

**Methods:**

An economic analysis of dapagliflozin versus metformin was conducted by using the Chinese Outcomes Model for T2DM with a time horizon of lifetime, which was developed and validated based on the Chinese population. The efficacy data of lowering HbA1c of dapagliflozin and metformin was derived from a network meta-analysis. Other clinical, cost and utility inputs were obtained from published sources. Lifetime discounted quality-adjusted life-years, cost, and incremental cost-effectiveness ratio were measured. The uncertainty was facilitated by one-way and probabilistic sensitivity analyses.

**Results:**

The comparison of metformin and dapagliflozin in Chinese patients with insufficient glycemic control by diet and exercise showed that dapagliflozin was more costly and produced fewer health benefits in our simulated cohort. The sensitivity analyses indicated that the results were robust.

**Conclusions:**

Dapagliflozin is not likely to be cost-effective compared with metformin for Chinese patients with T2DM inadequately controlled with diet and exercise.

## Introduction

Due to population growth and aging, the Global Burden of Disease Study showed that all-age disability-adjusted life-years (DALYs) for diabetes in 2016 were more than 57 million, which increased by 24.4% (95% CI 22.7–26.2) from 1990 to 2016 [1]. A recent study also showed that China has a large diabetes burden: one in four people with diabetes worldwide lives in China, where 10.9% of adults have diabetes and 35.7% have prediabetes [2, 3]. The entire Chinese economic burden from diabetes increased from 2.216 billion Chinese yuan in 1993 to 200 billion Chinese yuan in 2007 [4, 5].

The sodium-glucose co-transporter 2 (SGLT2) inhibitors are a new class of oral antidiabetic drugs (OAD) that act by reducing the reabsorption of renal-filtered glucose back into the bloodstream, thereby resulting in loss of glucose in the urine [6]. Several drugs in this class, such as dapagliflozin, canagliflozin and empagliflozin, have shown their favorable clinical efficacy [7]. In China, dapagliflozin has not been reimbursed. The recent two economic reports have suggested the dapagliflozin might be a cost-effective alternative compared with acarbose and glimepiride as monotherapy in drug-naive Chinese T2DM patients [8, 9]. However, both of them did not consider the metformin as a baseline comparator, which was recommended as the initial monotherapy for newly diagnosed T2DM by the latest Chinese guideline [10]. One latest economic report found dapagliflozin treatment was more cost-effective compared with metformin treatment for Chinese type 2 diabetes patients. However, this findings are largely driven by the effects of favorable weight profile on clinical, utility, and costs in the Cardiff model, which is based on the Western population [11].

Reports of a clinical benefit from dapagliflozin therapy in clinical trials caused great excitement among both endocrinologists and patients. The dapagliflozin monotherapy is well tolerated and effective in reducing the level of HbA1c, FPG, and body weight in patients with T2DM without increasing hypoglycaemia [12–14], which are the risk predictors of cardiovascular disease in patients with T2DM. However, the widespread use of dapagliflozin comes with a dramatic increase in health care costs compared with metformin, which is of concern to clinicians and payers. The need for the precise economic evaluation of dapagliflozin consumption in the Chinese context is becoming urgent. By employing our recently developed and validated Chinese Outcomes Model for T2DM (COMT) [15], the aim of this analysis was to provide economic evidence of using dapagliflozin monotherapy and metformin monotherapy as first-line therapy for Chinese adult patients with T2DM inadequately controlled by diet and exercise.

## Methods

### Model overview

**Fig. 1:**
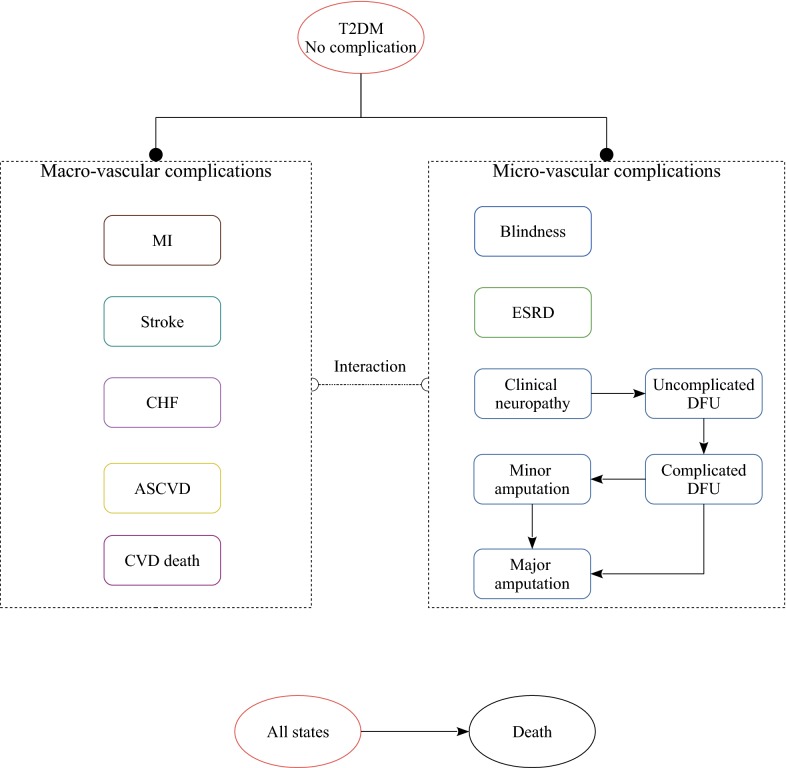
The structure of Chinese T2DM health policy model. *MI* myocardial infarction, *CHF* congestive heart failure, *CVD* cardiovascular disease, *ESRD* end-stage renal disease, *DFU* diabetic foot ulcer, *T2DM* type 2 diabetes mellitus

This study provides an economic assessment of dapagliflozin monotherapy for T2DM patients with inadequate glycemic control on diet and exercise. Patients would be assigned to metformin monotherapy or dapagliflozin monotherapy strategy. The analysis was carried out using the COMT [15, 16], a validated Chinese diabetes policy analysis model that would track several key diabetic macro- and micro-vascular complications for one hypothetical T2DM patient, including myocardial infarction (MI), congestive heart failure (CHF), cardiovascular disease (CVD), stroke, blindness, end-stage renal disease (ESRD), clinical neuropathy, foot ulcer, minor and major amputation (Fig. 1). The all-cause mortality would be adjusted based on the treatment effect and disease status. Each diabetic complication is an independent sub-model that was integrated with the COMT model. The transition probabilities of the model were estimated according to the latest Risk Equations for Complications of Type 2 Diabetes (RECODe) [17], which is adjusted validated based on the Chinese patient characteristics of T2DM. The details about the model development and validation could be found in our previous report [15]. During the model simulation, interconnectivity and interaction among sub-models of individual complication were permitted to allow the complication risks to be updated by using tracker parameters. The clinical and demographic characteristics of the hypothetical cohorts with T2DM were used for determining the annual disease progression: sex, age, smoking status, systolic blood pressure (SBP), glycated haemoglobin (HbA1c), total and high-density lipoprotein (HDL) cholesterol levels, serum creatinine, urine albumin:creatinine ratio, history of cardiovascular disease, use of antihypertensive, anticoagulant medications, statin and oral diabetes medication. During the simulation, risk parameters might be updated based on the treatment transition, thereby resulting in the likelihood of complication incidence. HbA1c, SBP, and cholesterol would worsen over time. More details about the model process could be found in our previous work [15]. The design of the model was the same for dapagliflozin and metformin strategy, with only risks of developing complications adjusted by the use of different treatments.

In line with most of the economic studies on T2DM [18], intervention, health and economic outcomes including costs, complication probabilities, life years and quality-adjusted life years (QALYs) were projected over a lifetime horizon in the current analysis. Costs and QALY were discounted at 5% annually, based on Chinese health economic recommendation [19]. When the incremental cost-effectiveness ratios (ICERs) were lower than the three times of per capita gross domestic product (GDP) of China in 2017 ($27,351), cost-effectiveness was assumed [19]. This economic study was based on a literature review and model techniques, and did not require approval by the Institutional Research Ethics Board.

### Clinical parameters

The treatment efficacy of dapagliflozin monotherapy or metformin monotherapy versus placebo on HbA1c was extracted from one recently published network meta-analysis reported efficacy, which included 75 randomized controlled trials involving 33,830 patients [20]. Due to the absence of the reported endpoints of SBP, total and HDL cholesterol between the dapagliflozin monotherapy and metformin monotherapy in this network meta-analysis [20], we conducted a new network meta-analysis for estimating these missing model inputs. In the literature review by searching PubMed, Web of Knowledge, no head-to-head comparisons of metformin and dapagliflozin was found to report the systolic blood pressure (SBP), total and HDL cholesterol. Thus, the data from an indirect comparison was used for synthesizing the treatment efficacy and safety inputs of the model by using network meta-analysis [21]. After examination of full-text articles by searching Medline, Embase, and the Cochrane database between 1990.01.01 and 2017.12.31, three randomized, placebo-controlled double-blind trials were eligible for estimating the efficacy of dapagliflozin monotherapy or metformin monotherapy versus placebo on SBP, total and HDL cholesterol [12–14]. In this indirect comparison, the placebo arm was used as a reference. By using the random effects model with mean difference as the summary measure [22], the network meta-analysis estimated the mean absolute changes from baseline in HbA1c levels, SBP, total and HDL cholesterol, which were employed in the 1st year of treatment (Table 1). In the subsequent year, HbA1c was simulated to rise naturally (nonlinear fashion), due to the progressive nature of the disease, according to the HbA1c trajectories analysis [23]. Similar assumptions were made for SBP, total and HDL cholesterol.

**Table 1:** Clinical parameters used in the model

Parameters	Metformin (versus placebo)		Dapagliflozin (versus placebo)		Source^a^
	Expected value	Range	Expected value	Range	
Decrease in HbA1c	1.13	0.9–1.37	0.50	0.21–0.78	[20]
Decrease in SBP (mmHg)	2.00	− 7.26 to 11.21	8.11	1.48–14.74	[12–14]
Decrease in total-C (mg/dL)	11.93	− 0.79 to 24.07	2.80	− 5.92 to 11.63	[12–14]
Increase in HDL-C (mg/dL)	2.01	− 7.67 to 11.53	8.10	1.24–15.14	[12–14]

*HbA1c* glycosylated hemoglobin, *HDL-C* high-density lipoprotein cholesterol, *SBP* systolic blood pressure

^a^The reported data from trials were synthesized by using network meta-analysis

In the simulation of the treatment sequence, patients were initially assigned to dapagliflozin or metformin and kept on the respective oral therapies until their HbA1c exceeds a pre-specified threshold (switching threshold); at which point he next therapy (rescue therapy) will be administered for patients in both arms. According to local clinical expert opinion, the HbA1c threshold for the treatment switch was defined at 8% and after reaching this, other antidiuretic regimens would be initiated and continued for the end of model simulation. The probabilities of hypoglycemia in dapagliflozin and metformin monotherapies were 1.1% and 9.1%, respectively, which were derived from the previous reports [8, 24].

The included T2DM Chinese patients were who failed to achieve adequate glucose control following diet and exercise and required drug treatments. The baseline characteristics and risk factor profiles were sourced from a recently published trial, which is a prospective phase III randomized controlled study and enrolled 393 Chinese patients with T2DM uncontrolled on diet and exercise [14]. The mean age was 51.3 years, and patients had a median disease duration of 0.2 years (Table 1). The proportion of males was 65.4%. The mean HbA1c level was 8.26%, and the mean baseline SBP was 123.7 mm Hg. In total, 40.5% of patients had a history of dyslipidemia and 38.9% had a history of hypertension. Patients were randomized to receive placebo (n = 132), dapagliflozin 5 mg (n = 128), or dapagliflozin 10 mg (n = 133) for 24 weeks. The model cohort was considered to be representative of Chinese patients who would be suitable to receive dapagliflozin as part of a Chinese treatment alternative. When data pertaining to a specific parameter that was used for estimating the complications [17], such as a history of smoking and anticoagulation usage, was not available, information from Chinese national cross-sectional studies was used as a Refs. [2, 3, 25].

### Costs and utilities

The present study was performed from the Chinese perspective of the healthcare services provider and only direct medical costs were considered in the model (Table 2). All cost data were presented in the 2017 US dollar ($). For dapagliflozin and metformin, the costs were calculated according to the 10 mg and 1500 mg daily dosage regimes, respectively. The price of dapagliflozin and metformin was derived from the study reported by Shao et al. [8], who collected the data from the Official drug price list of Price Bureau of China. After the first-line metformin and dapagliflozin monotherapy failed, patients would receive the rescue therapy based on the Chinese guideline of managing T2DM [10]. The annually costs of medicine and glucose testing strips were estimated from a large national population-based screening study [26], which interviewed 1482 adults with diabetes at 12 sites in China. Apart from the treatment cost of hyperglycemia, other potential direct health resource utilization, such as the costs of hospitalization and outpatient visits due to their developed complications, also reflected in the simulation, which was extracted directly from published literature or other local sources [4, 8, 9, 27–29]. The cost related to hypoglycemia were estimated from the patient records of two hospitals (Yueyang and Ren JiHospital) in 2016, including 172 patients diagnosed with hypoglycemia who visited the emergency clinic.

**Table 2:** Costs (2017 US $) and Health state utilities

Parameters	Expected value	Range	Source
Costs ($)			
Metformin 1500 mg per day	0.7	0.1–0.7	[8]
Dapagliflozin 10 mg per day	2.5	1.3–2.5	[8]
Anti-diabetic therapy per day (disease duration ≤ 3 year)	0.5	0.2–1.3	[26]
Anti-diabetic therapy per day (3 < disease duration ≤ 5 year)	0.8	0.2–1.7	[26]
Anti-diabetic therapy per day (6 ≤ disease duration < 10 year)	1.2	0.3–2.5	[26]
Anti-diabetic therapy per day (disease duration ≥ 10 year)	2.0	0.7–3.2	[26]
MI hospitalization per event	7383.0	6505.2–8260.9	[4, 8, 9, 27]
Care after MI per year	455.4	288.6–622.2	[4, 8, 9, 27]
Stroke hospitalization per event	2875.2	2184.6–4738.3	[4, 8, 9, 27]
Care after stroke per year	506.9	445.9–828	[4, 8, 9, 27]
CHF per year	1507.7	1254.6–2632.3	[4, 8, 9, 27]
ESRD per year	13,803.2	13,153.8–14,569.2	[29]
Blindness per year	1642.0	1430.4–1853.5	[4, 8, 9, 27]
Clinical neuropathy per month	60.9	26.2–101.4	[28]
Uncomplicated DFU per event	76.2	0–226.2	[28]
Complicated DFU per event	2293.3	1228.5–2880.8	[28]
Minor amputation per event	3316.9	2165.2–5038.9	[28]
Major amputation per event	5019.2	2981.1–7738.2	[28]
Care after major amputation per month	338.1	0–600.7	[28]
Hypoglycemia per event	70.0	0–855.5	Local charge
Utility values			
T2DM without complications	0.876	0.736–1	[30]
Utility decrements			
MI hospitalization for 1 month	1.000	0.236–1	[31]
MI after discharge	0.236	0.026–0.446	[30]
Stroke hospitalization for 1 month	1.000	0.326–1	[31]
Stroke after discharge	0.326	0.036–0.616	[30]
CHF	0.236	0.026–0.446	[30]
ESRD	0.400	0.19–0.61	[33]
Blindness	0.157	0.007–0.307	[33]
Clinical neuropathy	0.185	0.015–0.355	[30]
Uncomplicated DFU	0.250	0.213–0.287	[32]
Complicated DFU	0.300	0.165–0.435	[32]
Minor amputation	0.320	0.204–0.436	[32]
Major amputation	0.380	0.264–0.496	[32]

*MI* myocardial infarction, *CHF* congestive heart failure, *CVD* cardiovascular disease, *ESRD* end-stage renal disease, *DFU* diabetic foot ulcer, *T2DM* type 2 diabetes mellitus

Health state utility values (Table 2) were retrieved from a recent study, which enrolled 289 T2DM patients in China and determined health-state utility values of diabetes, neuropathy, heart disease and cerebrovascular disease by using EQ-5D-5L [30]. The decrement values related to MI and stroke hospitalization were assumed to be 1 as our previous study did because these patients would be in coma and bedridden [31]. Other utility values that were not included by this report, such as the utility values of ESRD and amputation, were derived from previous studies [32, 33].

### Sensitivity analyses

To examine the potential drivers of economic outcomes we carried out both one-way and probabilistic sensitivity analyses (PSA). In one-way sensitivity analyses, the incremental net-health benefit (INHB) would be used because the statistical inference of ICER is often difficult and INHB is a linear transformation of incremental costs and effectiveness. INHB calculated based on the following formula:

$${\text{INHB}}\left( \lambda \right) = \left( {\upmu_{\text{E1}} -\upmu_{{{\text{E}}0}} } \right) - \left( {\upmu_{\text{C1}} -\upmu_{{{\text{C}}0}} } \right)/\uplambda = \Delta {\text{E}} - \Delta {\text{C}}/\uplambda,$$

where μ_Ci_ and μ_Ei_ were the cost and effectiveness of treatment (i = 1) or control (i = 0), respectively, and λ was the three times of GDP per capita in 2017 [34]. The parameters and values were varied in the one-way sensitivity analysis, whose ranges were derived from the reported upper and lower 95% confidence intervals (Table 2). If no relevant data was available, an assumed range from 75 to 125% of base case values were used. For the PSA, probability distributions, were attached to all parameter in order to run second-order Monte-Carlo simulations (1000 iterations). The probability, proportions, utility value and utility decrements were modeled with beta distribution, cost with a triangle distribution, hazard ratio and patients characteristic profile with a normal distribution. If no standard error existed, then it was assumed to be 25% of the reported base case value. Based on the results of PSA, cost-effectiveness acceptability curve (CEAC) was produced.

## Results

### Base-case analysis

Compared with metformin monotherapy, dapagliflozin was associated with lower life expectancy and lower quality adjusted life expectancy (incremental life years: − 0.15; incremental QALYs: − 0.10), and an additional cost of $2188; leading to a dominated result (more expensive and fewer health benefits). These health detriments in the dapagliflozin treatment arm were driven by the increased cumulative incidence of MI, stroke, CHF, ESRD, Blindness, clinical neuropathy, minor and major amputation (Table 3).

**Table 3:** Base-case results for dapagliflozin compared to metformin

Outcomes	Metformin	Dapagliflozin	Difference^a^
Events			
MI	10.90%	11.01%	0.11%
Stroke	24.57%	25.40%	0.83%
CHF	15.56%	15.60%	0.03%
ESRD	4.748%	4.846%	0.097%
Blindness	5.17%	5.15%	− 0.02%
Clinical neuropathy	15.96%	16.00%	0.04%
Minor amputation	13.959%	13.963%	0.004%
Major amputation	10.501%	10.504%	0.003%
Total QALY	11.12	11.03	− 0.10
Total LY	24.86	24.71	− 0.15
Total cost (US $)	15,262	17,450	2188
ICER (US $/QALY)	NA	Dominated	

*MI* myocardial infarction, *CHF* congestive heart failure, *CVD* cardiovascular disease, *ESRD* end-stage renal disease, *DFU* diabetic foot ulcer, *QALY* quality-adjusted life year, *ICER* incremental cost-effectiveness ratio

^a^Compared with the control strategy (Metformin)

### Sensitivity analysis

**Fig. 2:**
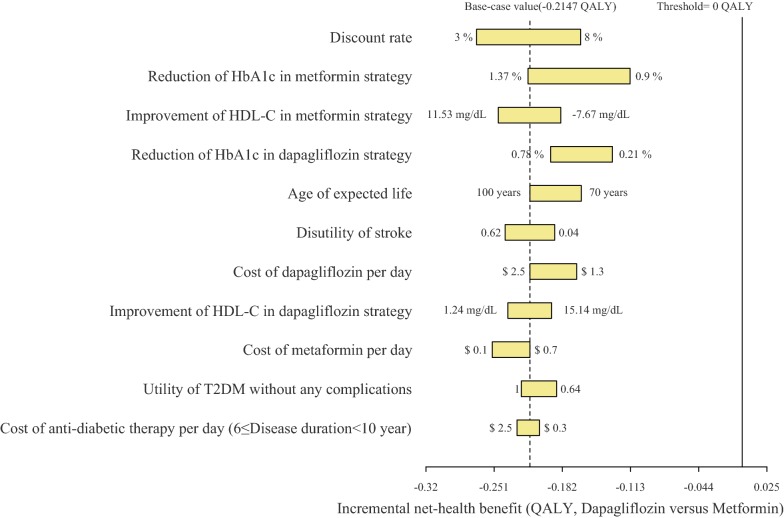
Tornado diagram representing the net health benefit in one-way sensitivity analysis for dapagliflozin versus metformin. The width of the bars represents the range of the results when the variables were changed. *HbA1c* glycosylated hemoglobin, *HDL-C* high-density lipoprotein cholesterol, *SBP* systolic blood pressure, *T2DM* type 2 diabetes mellitus

The one-way sensitivity analyses revealed that the results of the model were more sensitive to the discount rate because this variable was found to have the greatest impact on the INHB, which showed that the INHB of dapagliflozin versus metformin would be improved when it increased (Fig. 2). Other considerable sensitive variables were the treatment efficacy of metformin and dapagliflozin. However, none of the adjustments of parameters could push the net health benefit to exceed the threshold (0 QALY). The rest of the model inputs, such as the costs and utility values related to the complications, only had a paucity of impact on the model outcomes (The variations were lower than 10% of the base-case value).

**Fig. 3:**
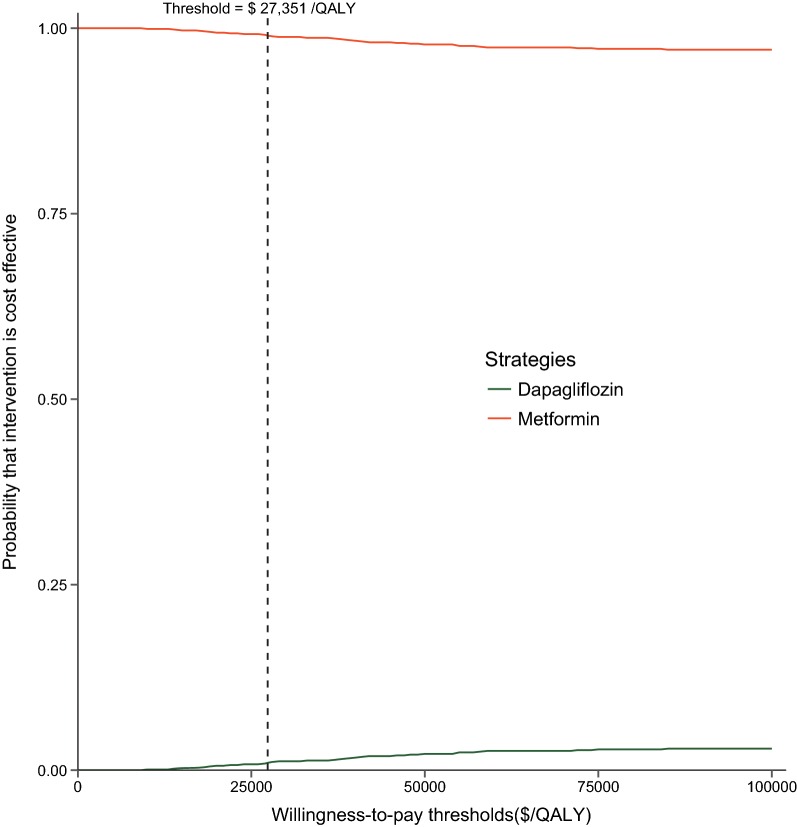
Cost-effectiveness acceptability curve for dapagliflozin versus metformin. *QALY* quality-adjusted life year, *ICER* incremental cost-effectiveness ratio

Based on the probabilistic sensitivity analyses, the cost-effectiveness acceptability curve (Fig. 3) showed the dapagliflozin strategy was associated with a 3% probability of being cost effective at the defined willingness-to-pay (WTP) threshold (three times GDP per capita of China in 2017).

## Discussion

The initiations of dapagliflozin and metformin monotherapy were associated with improvements in length and quality of life. However, the increased glycemic durability associated with metformin translated to more favorable health benefits gains compared with dapagliflozin. Due to the lower cost of metformin monotherapy, dapagliflozin was dominated. This economic finding strengthened the recommendation of Chinese guidelines that the preferred first-line drug for T2DM is metformin [10, 35]. One recently published meta-analysis also showed that metformin would be the most efficacious oral drugs for first-line monotherapy of T2DM in comparison with other oral glucose-lowering drugs, such as dapagliflozin and saxagliptin [20]. The latest economic evaluation by using the Cardiff Diabetes Model found the dapagliflozin monotherapy was more cost-effective compared with metformin monotherapy [11]. Except for the different simulation models, his discrepancy also contributed by the key model inputs. The efficacy of lowing HbA1c in metformin arm is superior to dapagliflozin in our analysis, which is derived from a recently published network meta-analysis. However, the findings of Cai et al. are largely driven by the effects of favorable weight profile on clinical, utility, and costs in the Cardiff model. However, the risk factors in diabetes, including the prevalence of obesity, are different between Asian and nonwhite populations [36].

One-way sensitivity analysis found the improvements in glycemic control observed in metformin compared with dapagliflozin strategy in the indirect comparison was a key driver of differentiation in terms of favorable cost-effectiveness profile. Although the net health benefit of dapagliflozin versus metformin was robust in all sensitivity analyses, the efficacy of anti-hyperglycemia (HbA1c levels) in dapagliflozin and metformin strategy had a considerable impact. When the HbA1c reduction of metformin strategy deceased to the 95% upper limit (0.632%), the net health benefit of metformin strategy would be improved. Due to multiple daily dosing and frequent GI side effects of metformin, the compliance with the metformin treatment would be incurred, which would lead to poor control and hence increased risk of the associated micro- and macro-vascular complications [37]. For improving the cost-effectiveness of metformin strategy, increasing the adherence to therapy is very important due to the chronic nature of diabetes.

This study is strengthened by employing the COMT model, which has manifested good model validity for established effects of medicines on surrogate endpoints such as glucose, blood pressure, BP, lipid profiles in the Chinese population. This economic analysis provides further evidence supporting the metformin as a primary option in first-line therapy for T2DM [38]. Previous studies have indicated that, in T2DM patients who were no longer responsive to diet and exercise, the dapagliflozin monotherapy was dominant compared with glimepiride and acarbose in the Chinese setting. Both of them indicated that weight control was the most influential factor affecting the economic outcome [8, 9].

There are several weaknesses in this study. Firstly, a Chinese perspective was adopted for costs and cost-effectiveness context (e.g., threshold and discount rates), which may affect the transferability of these findings in other regions. However, due to the transparent input profiles and treatment effects, region-specific cost and utility data could be input to replicate this evaluation to inform local decision-makers. Secondly, due to no direct comparison between dapagliflozin and metformin, an indirect comparison using placebo as a reference yielded more uncertainties around the model outcomes. One-way and sensitivity analyses showed the result was robust. Thirdly, as other cost-effectiveness analyses by using computer modeling techniques [18], this analysis extrapolated the lifetime clinical and economic outcomes beyond the trial follow-up period by translating short-term surrogate endpoints (risk factor profiles) to the incidence of diabetes-related complications and mortality. Therefore, the extrapolation of short-term data to a long-term horizon of metformin and dapagliflozin is a limitation, which should be addressed when explaining the finding. However, because our model was validated, the potential uncertainty of treatment should pertain to both metformin and dapagliflozin strategies which might not be considerably different. Finally, treatment effect data were extracted from clinic trials, which were substantially different from the real world [39], such as the non-adherence of medications. However, because the findings of this study reflected the Chinese common clinical conditions of managing T2DM, this study can provide relevant information for Chinese clinical and health policy decision-makers.

## Conclusion

This economic analysis found that metformin 1500 mg is likely to provide better health benefits at a lower cost than dapagliflozin 10 mg in monotherapy for Chinese patients with T2DM inadequately controlled with diet and exercise in the Chinese health care system. Therefore, our findings can inform physicians and patients when deciding on an optimal first line treatment for T2DM and can help policy makers in achieving more efficient allocation of health care resources for the management of T2DM in China.

## Data Availability

No additional data are available.
